# Radiomics with Clinical Data and [^18^F]FDG-PET for Differentiating Between Infected and Non-Infected Intracavitary Vascular (Endo)Grafts: A Proof-of-Concept Study

**DOI:** 10.3390/diagnostics15151944

**Published:** 2025-08-02

**Authors:** Gijs D. van Praagh, Francine Vos, Stijn Legtenberg, Marjan Wouthuyzen-Bakker, Ilse J. E. Kouijzer, Erik H. J. G. Aarntzen, Jean-Paul P. M. de Vries, Riemer H. J. A. Slart, Lejla Alic, Bhanu Sinha, Ben R. Saleem

**Affiliations:** 1 Department of Nuclear Medicine & Molecular Imaging, Medical Imaging Center, University Medical Center Groningen, University of Groningen, 9713 GZ Groningen, The Netherlands; 2 Department of Surgery, Division of Vascular Surgery, University Medical Center Groningen, University of Groningen, 9713 GZ Groningen, The Netherlands; 3 Department of Plastic Surgery, University Medical Center Groningen, University of Groningen, 9713 GZ Groningen, The Netherlands; 4 Department of Medical Microbiology and Infection Prevention, University Medical Center Groningen, University of Groningen, 9713 GZ Groningen, The Netherlandsb.sinha@umcg.nl (B.S.); 5 Department of Internal Medicine and Radboud Community for Infectious Diseases, Radboud University Medical Center, 6525 GA Nijmegen, The Netherlands; 6 Department of Medical Imaging, Radboud University Medical Center, 6525 GA Nijmegen, The Netherlands; 7 Department of Biomedical Photonic Imaging, University of Twente, 7522 NB Enschede, The Netherlands; 8 Magnetic Detection & Imaging Group, Technical Medical Centre, University of Twente, 7522 NB Enschede, The Netherlands

**Keywords:** intracavitary vascular (endo)graft infections, radiomics, machine learning, [^18^F]FDG PET, MAGIC criteria

## Abstract

**Objective:** We evaluated the feasibility of a machine-learning (ML) model based on clinical features and radiomics from [^18^F]FDG PET/CT images to differentiate between infected and non-infected intracavitary vascular grafts and endografts (iVGEI). **Methods:** Three ML models were developed: one based on pre-treatment criteria to diagnose a vascular graft infection (“*MAGIC-light* features”), another using radiomics features from diagnostic [^18^F]FDG-PET scans, and a third combining both datasets. The training set included 92 patients (72 iVGEI-positive, 20 iVGEI-negative), and the external test set included 20 iVGEI-positive and 12 iVGEI-negative patients. The abdominal aorta and iliac arteries in the PET/CT scans were automatically segmented using SEQUOIA and TotalSegmentator and manually adjusted, extracting 96 radiomics features. The best-performing models for the *MAGIC-light* features and *PET-radiomics* features were selected from 343 unique models. Most relevant features were combined to test three final models using ROC analysis, accuracy, sensitivity, and specificity. **Results:** The combined model achieved the highest AUC in the test set (mean ± SD: 0.91 ± 0.02) compared with the *MAGIC-light*-only model (0.85 ± 0.06) and the *PET-radiomics* model (0.73 ± 0.03). The combined model also achieved a higher accuracy (0.91 vs. 0.82) than the diagnosis based on all the MAGIC criteria and a comparable sensitivity and specificity (0.70 and 1.00 vs. 0.76 and 0.92, respectively) while providing diagnostic information at the initial presentation. The AUC for the combined model was significantly higher than the *PET-radiomics* model (*p* = 0.02 in the bootstrap test), while other comparisons were not statistically significant. **Conclusions:** This study demonstrated the potential of ML models in supporting diagnostic decision making for iVGEI. A combined model using pre-treatment clinical features and PET-radiomics features showed high diagnostic performance and specificity, potentially reducing overtreatment and enhancing patient outcomes.

## 1. Introduction

Intracavitary vascular graft and endograft infections (iVGEI) are relatively rare complications but are associated with high morbidity, mortality, and healthcare costs [1,2,3,4]. Therefore, diagnosis of iVGEI is of utmost importance, yet extremely difficult, currently relying on the “Management of Aortic Graft Infection Collaboration (MAGIC)” criteria [5]. However, given the diverse clinical presentation of iVGEI, these criteria are rather non-specific. With a specificity of 0.61, this poses a risk of overestimating the true diagnosis of iVGEI [6]. Additionally, the MAGIC criteria include a full diagnostic workup, including surgical signs. However, in this patient population—often critically ill and with multiple comorbidities—there is reluctance to perform surgery without confident diagnosis, and some patients may not even be candidates for surgery. This underscores the need for a diagnostic tool aiding early identification of iVGEI at the time of initial clinical presentation (“at-the-door situation”).

Imaging plays a pivotal role in diagnosing iVGEI. The current reference modality is computed tomography angiography (CTA), but the diagnostic accuracy is moderate, with a pooled sensitivity of 0.67 and specificity of 0.63 [5,7]. In contrast, 2-deoxy-2-[^18^F]fluoro-D-glucose positron emission tomography/computed tomography ([^18^F]FDG PET/CT) demonstrated high diagnostic performance in detecting iVGEI. In particular, visual uptake patterns had a strong diagnostic accuracy, with a pooled sensitivity of 0.94 and specificity of 0.81 [8]. These results are promising but based on small patient cohorts. Moreover, visual assessment is prone to observer variability [9]. This raises the question of whether more objective quantification of the uptake patterns using [^18^F]FDG PET/CT may aid iVGEI diagnosis. Standardised molecular image quantification may improve diagnostic accuracy, reproducibility, and therapy monitoring [10].

Radiomics extracts and analyses quantitative features from medical images. It relies on the concept that medical images contain information that reflects underlying pathophysiology imperceptible to the human eye [11]. It has been used in iVGEI with promising results but with a small cohort, single-centre design, and without using the integrated part of a machine-learning (ML) model [12]. Radiomics with ML may reveal information distinguishing infected from non-infected grafts, enhancing patient diagnostics, monitoring, and comparison between patients.

This proof-of-concept study aims to develop a radiomics and machine-learning-based tool to aid in differentiating between infected and non-infected intracavitary vascular grafts or endografts at the time of the initial clinical presentation.

## 2. Methods

### 2.1. Study Population

**Figure 1:**
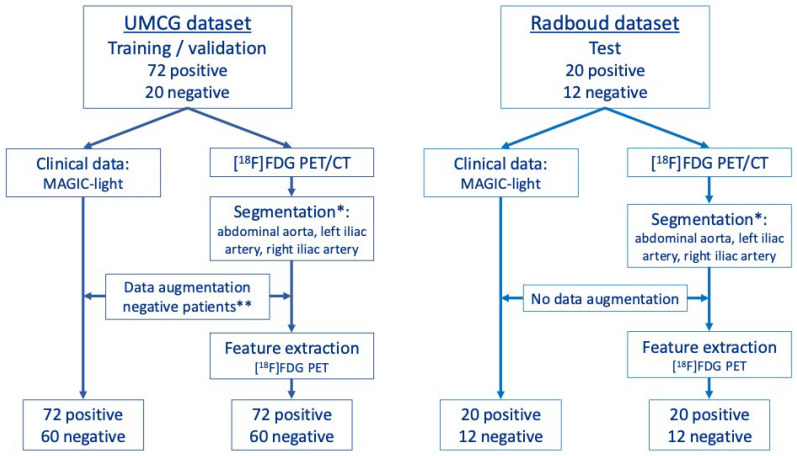
Flowchart of dataset composition and preprocessing for training, validation, and testing. * In both datasets, the abdominal aorta was automatically segmented using SEQUOIA [14], and the left and right iliac arteries were segmented using TotalSegmentator [15]. All segmentations were manually refined using Hermia Affinity Viewer (v4.0). The training and validation set was derived from the University Medical Center Groningen (UMCG) dataset, consisting of 72 positive cases and 20 original negative cases. ** To improve balance between positive and negative samples, data augmentation was applied to the negative group. In the MAGIC-light clinical dataset, missing data were imputed using the synthetic minority over-sampling technique (SMOTE). For radiomics data, features from [^18^F]FDG PET were extracted separately from each of the three segmented vessels—the abdominal aorta, left iliac artery, and right iliac artery—tripling the number of negative samples from 20 to 60. The test set consisted of data from Radboud University Medical Center, including 20 positive and 12 negative cases. No data augmentation was applied to the test set. For all positive cases and for the test set, the three vessels were considered as a single entity during radiomics feature extraction from [^18^F]FDG PET data.

This retrospective, multicentre study included two Dutch hospitals. At the University Medical Center Groningen (UMCG), 92 adult patients with suspected iVGEI who underwent diagnostic [^18^F]FDG PET/CT between 2002 and 2023 were included. Seventy-two patients were diagnosed with iVGEI based on positive intraoperative swabs or after multidisciplinary team decision based on the MAGIC criteria and clinical presentation. Twenty patients were labelled negative after multidisciplinary team decision (Figure 1).

At the Radboud University Medical Center (Radboudumc), 32 adult patients with a suspected iVGEI who underwent diagnostic [^18^F]FDG PET/CT scans between October 2013 and December 2018 were included. Twenty patients were diagnosed as positive using intraoperative swabs or multidisciplinary team decision, and twelve patients were labelled negative. The negative group also included patients with a vascular graft who underwent an [^18^F]FDG PET/CT scan for oncological indications, without the suspicion of iVGEI.

The Medical Research Involving Human Subjects Act obligation was waived by the local ethical committee of the UMCG (Research Register number: 202200389) and Radboudumc (CMO 2018-4512). Patient registries for opt-out checks revealed no objections. The data were stored and processed pseudonymised.

No minimum interval between graft placement and PET/CT imaging was applied in order to reflect real-world clinical scenarios and include diagnostically challenging cases with possible postoperative inflammation. The study adhered to the European Association of Nuclear Medicine (EANM) and the Society of Nuclear Medicine and Molecular Imaging (SNMMI) guidelines [13].

### 2.2. Machine-Learning Models of MAGIC-Light and PET-Radiomics Data

**Figure 2:**
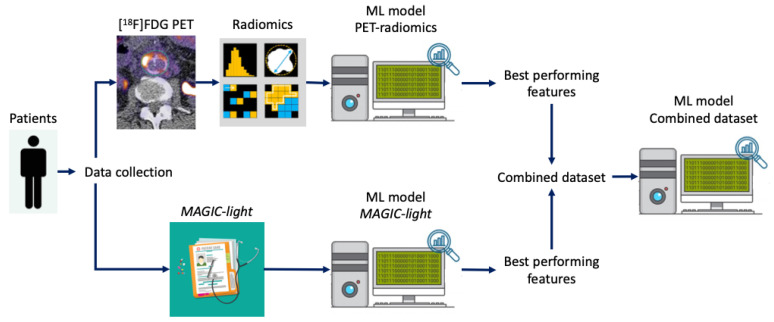
Composition of the three machine-learning (ML) models for iVGEI differentiation. Three ML models were developed: (1) a PET-radiomics model using features extracted from diagnostic [^18^F]FDG PET scans; (2) a MAGIC-light model based on pre-treatment clinical features; and (3) a combined model incorporating the most predictive features from both the PET-radiomics and MAGIC-light models. All models were trained using stratified 10-fold cross-validation.

Three ML models were developed: one based on pre-treatment MAGIC criteria (*MAGIC-light*), another using radiomics features from diagnostic [^18^F]FDG-PET scans (*PET-radiomics*), and one based on a combination of both datasets (Figure 2).

The MAGIC criteria are a standardised set of diagnostic criteria used to identify iVGEI [4]. Both the MAGIC criteria and [^18^F]FDG PET data were gathered preoperatively to simulate the clinical situation surgeons encounter when diagnosing patients before starting any treatment. Consequently, all MAGIC features, except for surgical findings, were included and are hereafter referred to as *MAGIC-light* features (Table 1). Each feature was collected from the electronic patient file system in binary format (1 = criterion present; 0 = absent or not performed). Additionally, the major and minor criteria were summed and added as features (Table S1).

### 2.3. Image Acquisition and Reconstruction

All [^18^F]FDG-PET/CT scans were acquired on integrated PET/CT systems (Biograph mCT 40/64-slice, Biograph Vision, or Biograph Vision Quadra, Siemens Healthineers, Knoxville, TN, USA) following the EANM guidelines [16]. The acquisition and reconstruction parameters are detailed in Table S2. PET images were reconstructed according to EANM Research Ltd. (EARL) (Vienna, Austria) guidelines to ensure reproducibility across systems and centres [17]. Low-dose CT scans were acquired for attenuation correction and anatomical localisation.

### 2.4. Segmentation

The abdominal aorta was automatically segmented using SEQUOIA [14]. The right and left iliac arteries, vertebrae T5-7, and spleen were automatically segmented using TotalSegmentator [15]. Due to the large anatomical variation of the vessels in this population, the segmentations were not always correct [14,15]. Therefore, the masks of the abdominal aorta and iliac arteries were manually adjusted in Hermia Affinity Viewer (version 4.0, Hermes Medical Solutions, Stockholm, Sweden) to include the entire vessel wall and [^18^F]FDG uptake associated with infection or inflammation while excluding any spill-over from neighbouring tissues, such as the ureters or the gut. The abdominal aorta was segmented from the diaphragm to the aortic bifurcation; the iliac arteries were segmented from the next slice to the inguinal ligament. Grafts in the groin exceeding the inguinal ligament were included, as this is often an important part of the infection. These segmentations were adjusted by two proficient MD observers with two to four years of experience in the field (FV, SL). Complex cases were reviewed and discussed with a vascular surgeon and a nuclear medicine physician experienced in the field of iVGEI (BRS, RHJAS). The masks of vertebrae T5-7 represented the bone marrow. When T5-7 were not visible on the CT scan or could not be delineated accurately, L2-4 were used instead. Together with the spleen, these masks were included for potential [^18^F]FDG uptake caused by immune system activity due to infection.

To measure interobserver variability and its impact on radiomics features, segmentations from ten randomly selected scans (five per observer) were independently manually adjusted by the second observer.

### 2.5. Data Augmentation

To address the data imbalance between iVGEI-positive and iVGEI-negative samples in the training data and reduce the chance of bias towards the majority class, data augmentation was applied specifically to the iVGEI-negative sample group. In the iVGEI-negative group, each of the three segmented vessels—the abdominal aorta, right iliac artery, and left iliac artery—were considered as independent entities for iVGEI-negative samples. This approach increased the number of negative samples from 20 to 60 (Figure 1). In contrast, features for iVGEI-positive samples were extracted from all three vessels combined as one entity. For the clinical data (*MAGIC-light* dataset [18]), the synthetic minority over-sampling technique (SMOTE) was applied to synthetically generate additional negative sample. The resulting *MAGIC-light* features were rounded to binary values and verified for clinical correctness by one of the observers (FV). No data augmentation was applied to the test set.

### 2.6. Radiomics Feature Extraction

Ninety-one features were extracted using the standardised framework for radiomics in python, pyRadiomics [19], in compliance with the Image Biomarker Standardisation Initiative (IBSI) guidelines [20]. The supplementary material provides a detailed explanation of feature extraction and a full list of extracted features. Additionally, SUV_mean_ and SUV_peak_ of the vessels, the bone marrow (average of three vertebrae masks), and spleen were included. Highly correlated radiomics features (Pearson correlation coefficient >0.9) were removed for robustness.

### 2.7. Machine-Learning Models

Seven feature selection methods, seven classifier algorithms, and seven numbers of selected features (*n* = 1–7) were combined to train and validate a total of (7 × 7 × 7=) 343 unique models. These feature selection methods and classifiers were selected based on literature use and computational efficiency [21]. A detailed list of the feature selection methods and classifiers can be found in the supplementary methods. Each model was trained using 10-fold cross-validation with 3 repetitions. The best-performing ML model for the *MAGIC-light* and *PET-radiomics* dataset was selected based on the highest average area under the receiver operating characteristic curve (AUC).

For the combined dataset, the selected features from the best-performing models of the individual datasets were utilised. Seven unique models (classifiers) were then trained using the combined dataset. The best-performing ML model was selected according to the highest average AUC from the cross-validation. Finally, the performance of the three final models was evaluated by the test set.

### 2.8. Statistical Analysis

Parametric outcomes are reported as mean ± standard deviation (SD) and non-parametric as median and interquartile range (IQR). Model performance was assessed by receiver operating characteristics (ROC) analysis on the independent test set from Radboudumc. Youden’s index determined optimal cut-off points to calculate accuracy, sensitivity, and specificity of the models. ROC curves were compared using DeLong test (*MAGIC-light* vs. *PET-radiomics*) and bootstrap test (combined model vs. others). The MAGIC “diagnosis” and “suspicion” of the test set were visually and descriptively added to the ROC. Suspicion was defined as one major or two minor criteria from different categories using only pre-treatment MAGIC criteria (*MAGIC-light*), and diagnosis was defined as at least one major criterion and any other criterion from a different category using all MAGIC criteria (Table S1). The most important features were defined as the selected features from the feature selection models in every fold. Feature importance was derived from model coefficients in every fold. The most important *PET-radiomics* features were tested for normality, followed by Bonferroni-corrected t-test or Mann–Whitney U rank test. To assess the interobserver variability of the manually adjusted segmentations, the Dice similarity coefficient (DSC) was calculated between the masks of the two observers. Furthermore, from those two masks, the interclass correlation coefficient (ICC) estimates and their 95% confidence intervals (CIs) were calculated between the SUV_mean_ and SUV_peak_ of the vessels and the radiomics features used by the most optimal ML model. This was performed based on a single-rating, absolute agreement, two-way mixed-effects model. *p* < 0.05 was considered statistically significant. Statistical analyses were performed using Python or R: the ICC with pingouin (v0.5.4), Student’s t-test and Mann–Whitney U rank test with scipy (v1.10.1), and the ROC analysis with scikit-learn (v1.3.2) and pROC [22].

## 3. Results

### 3.1. ML Model

**Figure 3:**
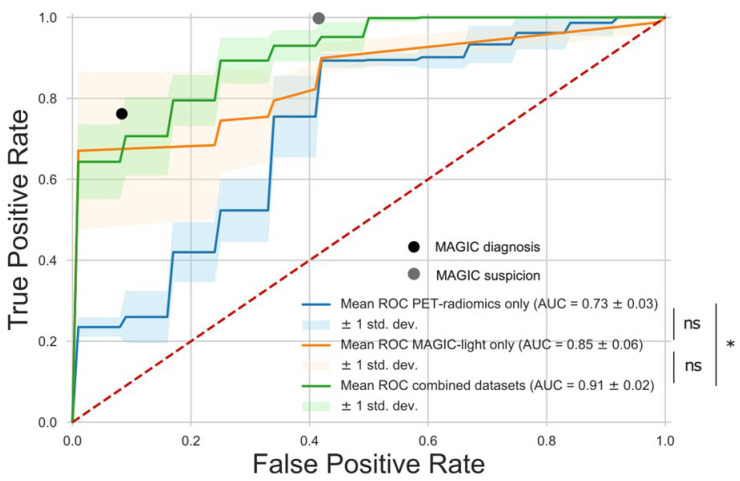
Receiver operating characteristic (ROC) curves of the three machine-learning (ML) models compared to MAGIC-based diagnostic performance. Mean ± standard deviation ROC curves on the test set are shown for the MAGIC-light model (orange), the PET-radiomics model (blue), and the combined model (green). The black dot represents the diagnostic performance based on the full MAGIC criteria (including surgical treatment criteria), while the grey dot reflects performance based on the pre-treatment MAGIC criteria (MAGIC-light), as defined by Lyons et al. [5] (Table S1). The combined ML model achieved sensitivity and specificity values comparable to those of the confirmed MAGIC diagnosis (0.70 and 1.00 vs. 0.76 and 0.92, respectively). The confirmed MAGIC diagnosis misses approximately 1 in 5 patients (76% sensitivity) but has high specificity (92%). In contrast, the suspected diagnosis (MAGIC-light) achieves 100% sensitivity but with lower specificity (58%), potentially leading to overtreatment. The combined model offers near-optimal performance, bridging the sensitivity–specificity gap between the two clinical approaches. It operates at the same stage as the MAGIC-light criteria while incorporating predictive power from radiomics features. * indicates statistically significant differences between ROC curves (*p* < 0.05); ns = not significant (*p* > 0.05).

The combined dataset model had the highest absolute AUC of 0.91 ± 0.02 (mean ± SD) on the test set and achieved higher accuracy in the test set (0.91 vs. 0.82) than a diagnosis based on all MAGIC criteria (the reference diagnostic workup, i.e., including surgical treatment criteria). As demonstrated in Figure 3 and Table 2, the sensitivity and specificity of the combined model were comparable to the MAGIC diagnosis (0.70 and 1.00 vs. 0.76 and 0.92, respectively). According to Figure 3 and Table 2, the MAGIC “diagnosis” (confirmed) misses approximately 1 out of 5 patients (76% sensitivity) and makes incorrect diagnosis in approximately 1 out of 10 patients (92% specificity). By contrast, the MAGIC “suspicion” (suspected) misses no patient (100% sensitivity) and makes incorrect diagnosis in approximately >4 out of 10 patients (58% specificity). However, this often occurs substantially earlier in the diagnostic process.

Comparing all three models, the AUC of the combined model was significantly higher compared to the *PET-radiomics*-only model (0.91 ± 0.02 vs. 0.73 ± 0.03; *p* = 0.02) but not compared to the *MAGIC-light*-only model (0.91 ± 0.02 vs. 0.85 ± 0.06; *p* = 0.43). No statistical difference was found between the *MAGIC-light* model and the *PET-radiomics* model (0.85 ± 0.06 vs. 0.73 ± 0.03; *p* = 0.30). The combined model resulted in the highest diagnostic accuracy (0.91 vs. 0.78 and 0.78) and the best combination of sensitivity and specificity (0.70 and 1.00 vs. 0.65 and 1.00 and vs. 0.90 and 0.58) compared to the *MAGIC-light*- and *PET-radiomics*-only models, respectively (Table 2).

The best-performing *MAGIC-light*-only model, based on the cross-validation from 343 models, was the combination of LASSO feature selection method and a random forest classifier using six selected features. The best-performing *PET-radiomics*-only model, based on the cross-validation from 343 models, was the combination of MRMRe feature selection method and a linear regression classifier using six selected features. The features used per fold were extracted from both models and combined to train another model. The best-performing model from the combined datasets, based on the cross-validation of seven models, was the XGBoost classifier.

### 3.2. Feature Importance

**Figure 4:**
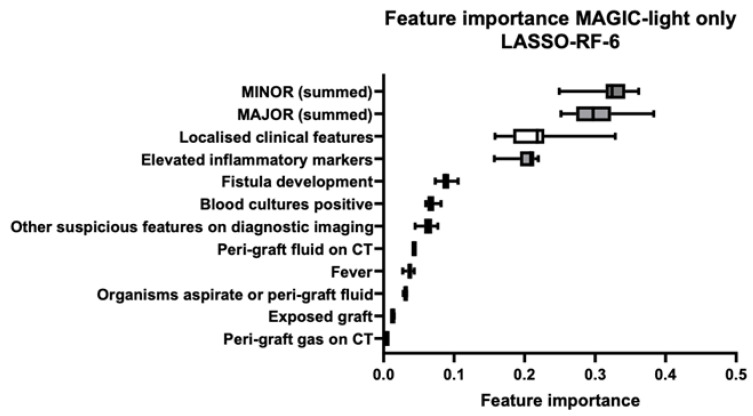
Feature importance in the final MAGIC-light model based on LASSO feature selection and random forest classification. The feature importance scores from the final MAGIC-light-only model are shown, which was developed using the least absolute shrinkage and selection operator (LASSO) for feature selection and a random forest (RF) classifier. Although the final model included six features, the boxplots display the distribution of feature importance scores across all training folds, which results in more than six features appearing. The most influential features included the summed minor and major MAGIC criteria, localised clinical features, and elevated inflammatory markers.

**Figure 5:**
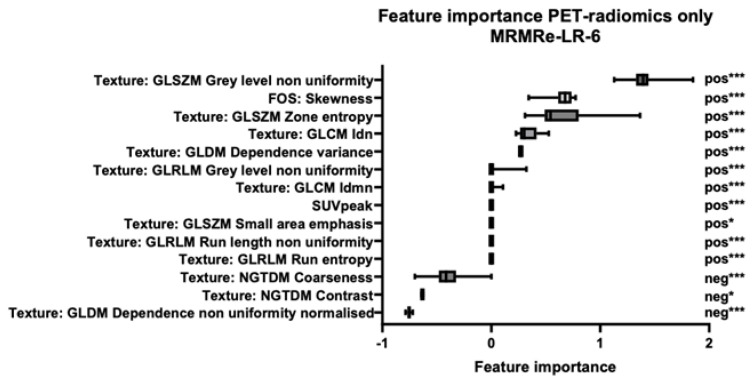
Feature importance in the final PET-radiomics model based on MRMRe feature selection and linear regression classification. The figure shows the feature importance scores from the final PET-radiomics-only model, developed using the minimum redundancy maximum relevance ensemble (MRMRe) for feature selection and a linear regression (LR) classifier. Although the final model included six features, the boxplots show the distribution of importance scores across all training folds, which results in more than six features appearing. The most predictive radiomic feature was GLSZM Grey-Level Non-Uniformity, which showed significantly higher values in the iVGEI-positive group (median: 38.6 vs. 12.0; *p* < 0.001). In contrast, GLDM Dependence Non-Uniformity Normalised was inversely associated with positive classification: higher values of this feature were associated with the iVGEI-negative group (median: 0.051 vs. 0.047; *p* < 0.001). FOS = first-order statistics; pos = feature values significantly higher in the positive group; neg = feature values significantly higher in the negative group. * = *p* < 0.05; *** = *p* < 0.001.

**Figure 6:**
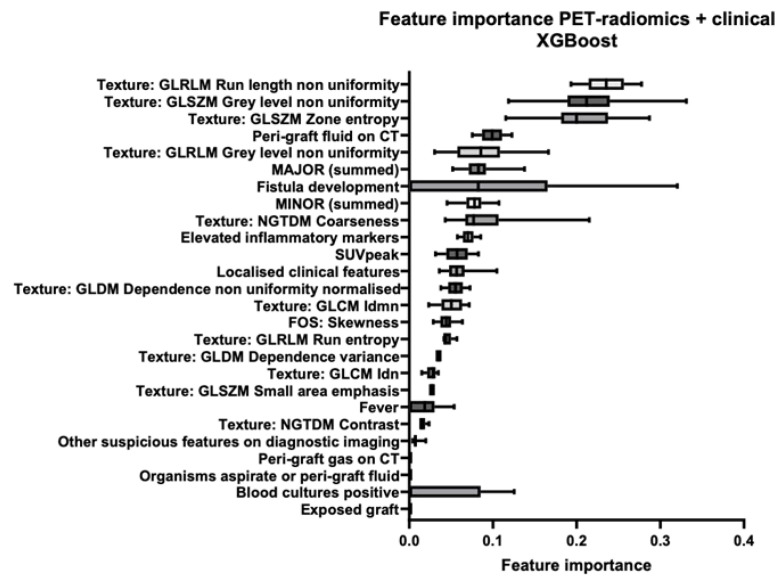
Feature importance in the final combined model using MAGIC-light and PET-radiomics features. The combined machine-learning model was developed using selected features from both the MAGIC-light- and PET-radiomics-only models, with classification performed using eXtreme gradient boosting (XGBoost). Although the final model included 12 features, the boxplots display the distribution of feature importance scores across all training folds, which results in more than 12 features appearing in the figure.

Figure 4, Figure 5 and Figure 6 illustrate the most important features and their distribution of importance of all folds from the three final models: *MAGIC-light*-only, *PET-radiomics*-only, and combined datasets, respectively. In the combined dataset model, four out of the five most important features were *PET-radiomics* features. The most important three were *GLRLM Run-Length Non-Uniformity*, *GLSZM Grey-Level Non-Uniformity*, and *GLSZM Zone Entropy*. In the supplementary material, a more detailed explanation of these features is given. The three most important *MAGIC-light* features were *peri-graft fluid on CT, summed major criteria*, and *fistula development*.

### 3.3. Interobserver Variability

The DSC between the manually adjusted masks of the two observers was 0.83 ± 0.06. Of all the extracted parameters and radiomics features, two ICCs and their 95% CIs were excellent, eight were good to excellent, five were moderate to excellent, and one was poor to excellent (Table 3).

## 4. Discussion

The findings of this proof-of-concept study demonstrate that an ML model integrating clinical features with PET-based radiomics holds significant promise for early and accurate diagnosis of iVGEI. This is evidenced by the model’s highest AUC and accuracy and maintained sensitivity and specificity comparable to diagnoses based on the full MAGIC criteria while offering additional diagnostic insights at the time of clinical presentation with suspected iVGEI (“at-the-door situation”). This underscores its potential as a powerful diagnostic tool. In contrast, a model relying solely on PET-radiomics features delivered less optimal results on an independent external dataset, reinforcing the critical role of combining clinical and imaging data to enhance diagnostic precision.

[^18^F]FDG PET/CT, currently a minor criterion of the MAGIC criteria, is an accepted diagnostic tool [5,7]. However, non-infected grafts often exhibit increased uptake postoperatively for an extended period due to the operation and/or foreign body reactions, complicating iVGEI diagnosis [23]. A meta-analysis demonstrated that the visual uptake pattern was most accurate in diagnosing iVGEI [8], prompting the question of whether quantifying [^18^F]FDG uptake patterns could enhance diagnostic accuracy. Standardised quantification of molecular images using objective methods such as radiomics may, however, lead to improved diagnostic accuracy, reproducibility, and therapy monitoring [10,11].

Our proposed quantitative method achieved higher accuracy than a diagnosis based on the full MAGIC criteria (slightly lower sensitivity but higher specificity). The suspicion group has a high sensitivity but drastically overestimates the true diagnosis, increasing the risk of unnecessary surgery and subsequent morbidity and mortality. With the current diagnostic tools (MAGIC), specificity can only be increased by performing surgery. In addition, a substantial proportion of this patient group is considered unfit for surgery. The presented combined model can bridge this gap and perform equally well as MAGIC but at the time of clinical presentation, even prior to (potential) surgery.

Future studies may apply all three models to a diverse range of patients with vascular grafts to assess their performance within a clinical context. Using ML is perhaps useful in the diagnostic and monitoring decision making, as was demonstrated for the most important features above [11]. An important next step would be to compare our method in a clinical setting with diagnoses made by nuclear medicine specialists to evaluate its added value in the context of [^18^F]FDG PET/CT imaging. We anticipate that the presented model will yield more robust outcomes due to its objective nature.

This study has several strengths to be underscored. First, this is the largest study population thus far comprising individuals suspected of and diagnosed with iVGEI who underwent an [^18^F]FDG PET/CT. Moreover, this is the first study to explore the feasibility of an ML model as a diagnostic tool for iVGEI. Radiomics features of [^18^F]FDG PET were utilised before for diagnosis of iVGEI [12]. However, only separate features were examined for predictive modelling, and no ML model was developed, which is an integrated part of radiomics. In addition, the study cohort was small, comprising only sixteen patients who had a suspicion of iVGEI. The present study is also unique in comparing the diagnostic accuracy of features from [^18^F]FDG PET with the diagnostic accuracy of clinical variables within the same timeframe in clinical decision making. Additionally, we now examined the added value of PET features in this decision-making process. In the Supplementary Materials, we further discuss the meaning of the most important radiomics features. A study by Mitra et al. compared the predictive value of [^18^F]FDG PET/CT outcome with other diagnostic tests in detecting iVGEI, i.e., white cell count, erythrocyte sedimentation rate, and C-reactive protein [24]. However, these inflammatory markers were only a minor part of the entire diagnostic workflow and were rather non-specific [5]. Another study by Dong et al. investigated the concordance of [^18^F]FDG PET/CT with MAGIC criteria in 35 patients, demonstrating a high concordance of 88.6% [25]. However, a definitive diagnosis was not utilised for comparative analysis, and the incremental benefit of [^18^F]FDG PET/CT in this context was not investigated. As an indirect comparison, the approach of using [^18^F]FDG PET/CT data for radiomics in diagnosing complex prosthetic material infections added to standard clinical decision criteria (modified Duke criteria) has been successfully applied for patients with suspected aortic prosthetic valve endocarditis in a recent study [26]. Another recent study investigated the use of an ML model in the clinical decision-making process using clinical data (modified Duke criteria) to diagnose prosthetic valve endocarditis [27]. To the best of our knowledge, our study is the first to investigate the approach of an ML model with radiomics in [^18^F]FDG PET, clinical data, and a combination of both.

This study has some limitations. First, the *MAGIC-light* criteria are part of the final diagnosis, which creates a confirmation bias, thereby enhancing the results of the *MAGIC-light* model. A positive culture of the explanted graft would be a better “gold standard”. Apart from not always being available (not all patients undergo surgery) and not being without risks, the entire process of micro-organism identification is complex and does not have optimal sensitivity and specificity [4]. Therefore, to obtain a large study population using positive culture as the diagnostic endpoint is difficult and would introduce a substantial recruitment bias. Second, [^18^F]FDG PET/CT was used in the minor features of the *MAGIC-light* features. This might have increased the outcome of the *MAGIC-light*-only model, as it was among the most crucial features. Hence, this also increased the importance of [^18^F]FDG PET in the combined dataset model. However, the increase in performance of the combined dataset strongly suggests that radiomics in [^18^F]FDG PET is of higher added value than its use solely as a minor criterion. Third, the radiomics model was trained and tested with data from a single vendor, with imbalanced scanner types, limiting robustness and generalisability. Future studies should include diverse centres, vendors, and scanner types. Fourth, the external test set had different reconstruction parameters, such as larger slice thickness, potentially affecting feature details. Radiomics is sensitive to variations in scanners, acquisition, and reconstruction methods, emphasising the need for standardisation [28]. Although 87% of the scans were acquired on the same scanner, and all reconstructions followed the EARL standards to ensure consistency, some residual bias may persist. Future studies could consider harmonisation techniques to further address inter-scanner variability, especially in multicentre settings. Further research could also explore comparisons between radiomics and deep learning, despite the reliance on large datasets in the latter, which poses challenges for rare diseases like iVGEI. Fifth, although this study included a relatively large cohort for iVGEI, the dataset remains small for ML applications. To address class imbalance, data augmentation was applied; however, this may introduce bias or alter underlying data distributions. Future work should look into multicentre datasets and more advanced data augmentation techniques to enhance robustness and generalisability. Sixth, not all patients in the external test set had a suspicion of iVGEI. This may have influenced the results, potentially resulting in higher specificity. Seventh, in most scans, manual adjustment of the automatically generated segmentations was needed due to the large anatomical variations in this study population. Therefore, features based on the shape of the segmentations could not be used. Despite moderate Dice scores of 0.83 ± 0.06, indicating imperfect mask overlap, the variability had minimal impact on critical features, which showed high correlation, suggesting that feature extraction is robust to small segmentation discrepancies. Last, only the abdominal aorta and iliac arteries were included in this study. Future studies should include vascular grafts in other surgical sites, as outcomes of infections in different surgical sites can differ [29].

To conclude, this proof-of-concept study demonstrated the potential of an ML model in the diagnostic decision-making process of iVGEI. The combination of clinical features and radiomics in PET resulted in the best performance compared to ML models based on either one of the two datasets, and it holds promising potential for diagnosing iVGEI during the early diagnostic phase. These positive findings set the stage for large-scale, prospective, multicentre studies, including multivendor datasets aiming to develop a robust ML model for routine use in daily clinical practice.

## Figures and Tables

**Table 1:** MAGIC-light features. Every feature was collected binary, where one denoted “the criterion was present in this patient”, and zero indicated “the criterion was not present in this patient” or “the test was not performed in this patient”. All pre-treatment MAGIC criteria were collected as described by Lyons et al. [5].

Clinical		Radiology		Laboratory		
Major Criteria	Minor Criteria	Major Criteria	Minor Criteria	Major Criteria	Minor Criteria	
Graft insertion in infected site	Localised clinical features	Peri-graft fluid on CT	Other suspicious features on diagnostic imaging	Organisms recovered from percutaneous aspirate	Elevated inflammatory markers	Summed major criteria
Exposed graft	Fever	Peri-graft gas on CT			Positive blood cultures	Summed minor criteria
Fistula development		Increase in peri-graft gas volume				

**Table 2:** Performance of the three final models measured in area under the receiver operating characteristic curve (AUC) ± standard deviation (SD). Furthermore, after calculating Youden’s index for the optimal cut-off point, accuracy, sensitivity, and specificity were calculated. The first row is the result of a diagnosis based on the full MAGIC criteria (i.e., including treatment and post-treatment criteria), as set out by Lyons et al. [5] Highest values of every variable are highlighted in bold.

Model	AUC ± SD	Accuracy	Sensitivity	Specificity
MAGIC diagnosis	-	0.82	0.76	0.92
MAGIC suspicion	-	0.85	**1.00**	0.58
*MAGIC-light*-only	0.85 ± 0.06	0.78	0.65	**1.00**
*PET-radiomics*-only	0.73 ± 0.03	0.78	0.90	0.58
Combined	**0.91 ± 0.02**	**0.91**	0.70	**1.00**

**Table 3:** Intraclass correlation coefficient (ICC) estimates and their 95% confidence intervals (CIs) between manually adjusted masks from two observers. This was calculated for volume, SUV_mean_, SUV_peak_, and the features that appeared most important from the PET-radiomics-only model. Based on the 95% confidence interval of the ICC estimate, values less than 0.5, between 0.5 and 0.75, between 0.75 and 0.9, and greater than 0.90 were considered of poor, moderate, good, and excellent reliability, respectively.

Feature	ICC	95% CI
SUV_mean_	0.99	[0.96 1.00]
Texture: GLDM Dependence Variance	0.99	[0.95 1.00]
Texture: GLSZM Zone Entropy	0.97	[0.89 0.99]
Texture: GLRLM Run Entropy	0.97	[0.88 0.99]
Volume	0.97	[0.87 0.99]
Texture: NGTDM Contrast	0.96	[0.86 0.99]
SUV_peak_	0.96	[0.83 0.99]
FOS: Skewness	0.96	[0.83 0.99]
Texture: NGTDM Coarseness	0.95	[0.82 0.99]
Texture: GLRLM Run-Length Non-Uniformity	0.94	[0.79 0.99]
Texture: GLDM Dependence Non-Uniformity Normalised	0.92	[0.71 0.98]
Texture: GLRLM Grey-Level Non-Uniformity	0.92	[0.71 0.98]
Texture: GLCM IDN	0.91	[0.69 0.98]
Texture: GLSZM Grey-Level Non-Uniformity	0.90	[0.66 0.98]
Texture: GLCM IDMN	0.88	[0.59 0.97]
Texture: GLSZM Small Area Emphasis	0.62	[0.04 0.89]

ICC = Mean Intraclass Correlation Coefficient; 95% CI = 95% Confidence Interval; SUV = Standardised Uptake Value; GLDM = Grey-Level Dependence Matrix; GLSZM = Grey-Level Size Zone Matrix; GLRLM = Grey-Level Run-Length Matrix; NGTDM = Neighbouring Grey Tone Difference Matrix; FOS = First-Order Statistics; GLCM = Grey-Level Co-Occurrence Matrix.

## Data Availability

The data supporting the findings of this study are available from the corresponding author upon reasonable request. The data are not publicly available due to privacy and ethical restrictions.

## Supplementary Materials

The following supporting information can be downloaded at: https://www.mdpi.com/article/10.3390/diagnostics15151944/s1, radiomics feature extraction, extra discussion radiomics features, Table S1: MAGIC vs MAGIC-light criteria, Table S2: Acquisition and reconstruction parameters 2-deoxy-2-[^18^F]fluoro-D-glucose positron emission tomography/computed tomography.
