# Supplementary material for: Radiomics with Clinical Data and [18F]FDG-PET for Differentiating Between Infected and Non-Infected Intracavitary Vascular (Endo)Grafts: A Proof-of-Concept Study
Source: Diagnostics (Basel). 2025 Aug 2;15(15):1944. doi: 10.3390/diagnostics15151944 (PMC12346819; doi:10.3390/diagnostics15151944)
Supplement: Supplementary file 1 [file diagnostics-15-01944-s001.zip › diagnostics-3760578-supplementary.pdf]

Supplementary

Supplementary methods

Radiomics feature extraction

The PET images were converted from activity (Bq/mL) into standardised uptake values (SUV). PET voxels were resampled to isotropic voxel spacing of 2.0x2.0x2.0 mm^3 using cubic B-Spline interpolation. These features include first order statistics, *Grey Level Co-occurrence Matrix* (*GLCM*), *Grey Level Run Length Matrix* (*GLRLM*), *Grey Level Size Zone Matrix* (*GLSZM*), *Neighbouring Grey Tone Difference Matrix* (*NGTDM*), *Grey Level Dependence Matrix* (*GLDM*). Shape features were excluded due to their sensitivity to manual segmentation and data augmentation. Missing values in the dataset were imputed with the mean statistic for numeric data or the most frequent statistic for binary data. Numeric features were normalised using z-normalisation, and the summed major and minor features were min-max normalised.

Extracted features

First-order statistics

- 10 Percentile
- 90 Percentile
- Energy
- Entropy
- Interquartile Range
- Kurtosis
- Maximum
- Mean Absolute Deviation
- Mean
- Median
- Minimum
- Range
- Robust Mean Absolute Deviation
- Root Mean Squared
- Skewness
- Total Energy
- Uniformity
- Variance

GLCM

- Autocorrelation
- Cluster Prominence
- Cluster Shade
- Cluster Tendency
- Contrast

- Correlation
- Difference Average
- Difference Entropy
- Difference Variance
- Id
- Idm
- Idmn
- Idn
- Imc1
- Imc2
- Inverse Variance
- Joint Average
- Joint Energy
- Joint Entropy
- MCC
- Maximum Probability
- Sum Average
- Sum Entropy
- Sum Squares

GLDM

- Dependence Entropy
- Dependence Non Uniformity
- Dependence Non Uniformity Normalised
- Dependence Variance
- Grey Level Non Uniformity

- Grey Level Variance
- High Grey Level Emphasis
- Large Dependence Emphasis
- Large Dependence High Grey Level Emphasis
- Large Dependence Low Grey Level Emphasis
- Low Grey Level Emphasis
- Small Dependence Emphasis
- Small Dependence High Grey Level Emphasis
- Small Dependence Low Grey Level Emphasis

GLRLM

- Grey Level Non Uniformity
- Grey Level Non Uniformity Normalised
- Grey Level Variance
- High Grey Level Run Emphasis
- Long Run Emphasis
- Long Run High Grey Level Emphasis
- Long Run Low Grey Level Emphasis
- Low Grey Level Run Emphasis
- Run Entropy
- Run Length Non Uniformity
- Run Length Non Uniformity Normalised
- Run Percentage
- Run Variance
- Short Run Emphasis
- Short Run High Grey Level Emphasis

- Short Run Low Grey Level Emphasis

GLSZM

- Grey Level Non Uniformity
- Grey Level Non Uniformity Normalised
- Grey Level Variance
- High Grey Level Zone Emphasis
- Large Area Emphasis
- Large Area High Grey Level Emphasis
- Large Area Low Grey Level Emphasis
- Low Grey Level Zone Emphasis
- Size Zone Non Uniformity
- Size Zone Non Uniformity Normalised
- Small Area Emphasis
- Small Area High Grey Level Emphasis
- Small Area Low Grey Level Emphasis
- Zone Entropy
- Zone Percentage
- Zone Variance

NGTDM

- Busyness
- Coarseness
- Complexity
- Contrast
- Strength

Quantitative features

- SUL_{mean} vessels

- SUL_{peak} vessels
- SUL_{mean} bone marrow
- SUL_{peak} bone marrow
- SUL_{mean} spleen
- SUL_{peak} spleen

The seven feature selection methods:

- Least absolute shrinkage and selection operator (LASSO);
- Support vector machines-recursive feature elimination (SVM-RFE);
- ReliefF;
- Minimum redundancy and maximum relevance ensemble (MRMRe);
- Mutual information (MI);
- t-score;
- ANOVA.

The seven classifiers:

- Logistic regression (LR);
- Support vector machine-radial basis function kernel (SVM-RBF);
- Random forest (RF);
- Extra tree classifier (ETC);
- K-nearest neighbour (KNN)
- Extreme gradient boosting (XGBoost);
- Neural network (NN).

Number of features:

- 1 to 7

Supplementary discussion radiomics features

In the *PET-radiomics*-only model, *GLSZM Grey Level Non-Uniformity* was the most important feature. A higher value of that feature – in this model contributing to a positive prediction – means more heterogeneity in intensity values. The third most important feature was the *GLSZM Zone Entropy*, which indicated more heterogeneous uptake patterns in positive cases. This is in concordance with literature on visual uptake, as focal hotspots are representative of infections, whereas diffuse, homogeneous uptake is more indicative of noninfected inflammatory processes[1,2]. *First Order Skewness*, the second most important feature, indicates a right-tailed uptake distribution for positive cases, which is probably due to the focal uptake spots compared to a more homogeneous uptake distribution in negative samples. Interestingly, in the combined model, the *GLRLM Run Length Non-Uniformity* was most important, whereas it had no importance in the *PET-radiomics* only model. The feature makes sense however, as these higher values signify a less uniform pattern, which indicates greater variability in how pixels of similar intensity values are distributed.

Supplementary references

1 Reinders Folmer EI, von Meijenfeldt GCI, te Riet ook genaamd Scholten RS, van der Laan MJ, Glaudemans AWJM, Slart RHJA, et al. A systematic review and meta-analysis of 18F-fluoro-d-deoxyglucose positron emission tomography interpretation methods in vascular graft and endograft infection. *J Vasc Surg* 2020;**72**(6):2174-2185.e2. Doi: 10.1016/j.jvs.2020.05.065.
2 Keidar Z, Pirmisashvili N, Leiderman M, Nitecki S, Israel O. 18F-FDG uptake in noninfected prosthetic vascular grafts: incidence, patterns, and changes over time. *J Nucl Med* 2014;**55**(3):392–5. Doi: 10.2967/jnumed.113.128173.

Supplementary Tables

Table S1: MAGIC vs MAGIC-light *criteria In this table, all MAGIC criteria are presented as described by Lyons et al. [5]. According to the MAGIC criteria, VGEI is suspected in the presence of one major or two minor criteria of the three different categories, and VGEI is diagnosed when there is at least a single major criterion and any other criterion from another category. The red crosses show which criteria were excluded from the MAGIC-light features to represent the pre-treatment criteria. The green circles represent the criteria added to the MAGIC-light features, that are not included in the original MAGIC criteria. In the original MAGIC criteria, aortic graft infection is suspected in a patient with any isolated major criterion, or minor criteria from two of the three categories: clinical/surgical, radiological, or laboratory. Aortic graft infection is diagnosed in the presence of a single major criterion, plus any other criterion (major or minor) from another category.*

**Clinical / Surgical**		**Radiology**		**Laboratory**		
**Major criteria**	**Minor criteria**	**Major criteria**	**Minor criteria**	**Major criteria**	**Minor criteria**	
Graft insertion in infected site	Localised clinical features	Peri-graft fluid on CT	Other suspicious features on diagnostic imaging	Organisms recovered from percutaneous aspirate	Elevated inflammatory markers	Summed major criteria
Exposed graft	Fever	Peri-graft gas on CT			Blood cultures positive	Summed minor criteria
Fistula development		Increase in peri-graft gas volume			Organisms from explanted graft	
Pus around the graft at surgery					Organisms from intra-operative specimen	

Table S2: Acquisition and reconstruction parameters 2-deoxy-2-[^{18}F]fluoro-D-glucose positron emission tomography/computed tomography. Numbers are presented as N (%).

	**UMCG – training / validation set (n=92)**		**Radboudumc – test set (n=32)**	
**Parameters**	**Positive**	**Negative**	**Positive**	**Negative**
Number of scans	72 (58%)	20 (16%)	20 (16%)	12 (10%)
**Scanners**				
Biograph mCT	72 (58%)	13 (10%)	20 (16%)	4 (3%)
Biograph Vision	0	6 (5%)	0	8 (6%)
Biograph Vision Quadra	0	1 (1%)	0	0
**Reconstruction kernel**				
Gaussian 5	13 (19%)	5 (4%)	0	2 (2%)
Gaussian 6	53 (43%)	10 (8%)	0	1 (1%)
Gaussian 7	0	4 (3%)	0	9 (7%)
Gaussian 8	6 (5%)	1 (1%)	20 (16%)	0
**Reconstruction method**				
PSF+TOF	66 (53%)	19 (15%)	20 (16%)	8 (6%)
OSEM3D	6 (5%)	1 (1%)	0	4 (3%)
**Slice thickness**				
2 mm	72 (58%)	20 (16%)	0	2 (2%)
3 mm	0	0	0	6 (5%)
5 mm	0	0	20 (16%)	4 (3%)
